# Capsaicin Supplementation Improved Risk Factors of Coronary Heart Disease in Individuals with Low HDL-C Levels

**DOI:** 10.3390/nu9091037

**Published:** 2017-09-20

**Authors:** Yu Qin, Li Ran, Jing Wang, Li Yu, He-Dong Lang, Xiao-Lan Wang, Man-Tian Mi, Jun-Dong Zhu

**Affiliations:** Chongqing Medical Nutrition Research Center, Chongqing Key Laboratory of Nutrition and Food Safety, Research Center for Nutrition and Food Safety, Institute of Military Preventive Medicine, Third Military Medical University, Chongqing 400038, China; yuer_forever@hotmail.com (Y.Q.); ranli1983@sina.com (L.R.); wangjingxi77577@sina.cn (J.W.); gyulilove.happy@gmail.com (L.Y.); lang0403@163.com (H.-D.L.); wxiaolan-006@163.com (X.-L.W.); mimantian@hotmail.com (M.-T.M.)

**Keywords:** capsaicin, low high-density lipoprotein cholesterol, lipids, phospholipid transfer protein, anti-inflammatory, clinical trial

## Abstract

Low high-density lipoprotein cholesterol (HDL-C) is associated with an increased risk of coronary heart disease (CHD). This study aimed to evaluate the effects of capsaicin intervention on the serum lipid profile in adults with low HDL-C. In a randomized, double-blind, controlled clinical trial, 42 eligible subjects were randomly assigned to the capsaicin (*n* = 21, 4 mg of capsaicin daily) or to the control group (*n* = 21, 0.05 mg of capsaicin daily) and consumed two capsaicin or control capsules, which contained the powder of the skin of different peppers, twice daily for three months. Thirty-five subjects completed the trial (18 in the capsaicin group and 17 in the control group). The baseline characteristics were similar between the two groups. Compared with the control group, fasting serum HDL-C levels significantly increased to 1.00 ± 0.13 mmol/L from 0.92 ± 0.13 mmol/L in the capsaicin group (*p* = 0.030), while levels of triglycerides and C-reactive protein and phospholipid transfer protein activity moderately decreased (all *p* < 0.05). Other lipids, apolipoproteins, glucose, and other parameters did not significantly change. In conclusion, capsaicin improved risk factors of CHD in individuals with low HDL-C and may contribute to the prevention and treatment of CHD.

## 1. Introduction

In the last few decades, many epidemiological studies indicated that serum low high-density lipoprotein cholesterol (HDL-C) with or without other lipid disorders was associated with an increased risk of coronary heart disease [1]. A previous meta-analysis found that the prevalence of overall low HDL-C (HDL<1.03 mmol/L in men and <1.30 mmol/L in women) and low HDL-C without other lipid disorders were higher among Asians (33.1% and 22.4%) compared with non-Asians (27.0% and 14.5%) [1]. Interestingly, a recent cross-sectional study showed a novel lower prevalence of overall low HDL-C and low HDL-C without other lipid disorders (21.1% and 10.5%) among populations in Chongqing, China [2], which may be partially attributed to the frequently intake of chili by these people. 

Chili is rich in capsaicin. Many animal and cellular experiments found that capsaicin performed some health benefits including having anti-inflammatory effects, decreasing plasma cholesterol levels, and promoting vascular and metabolic health [3,4,5,6]. For instance, dietary capsaicin effectively lowered plasma cholesterol and inhibited the formation of atherosclerotic plaque in hamsters and rats [7,8,9]. However, few human studies have researched the influences of capsaicin on vascular health or its risk factors. Our recent clinical trial found that capsaicin-containing chili ameliorated glucose and lipid disorders in women with gestational diabetes mellitus [10]. Based on the lower prevalence of low HDL-C in Chongqing and the health effect of capsaicin, we presumed that capsaicin may have an ameliorating effect on fasting serum HDL-C.

Cholesterylester transfer protein (CETP), phospholipid transfer protein (PLTP), and lecithin cholesterol acyltransferase (LCAT) are considered to play important roles in HDL-C metabolism and function in the process of atherogenesis [11,12]. Additionally, inflammation is generally associated with HDL-C metabolism [13]. Therefore, capsaicin may alter HDL-C through its effects on the above proteins and inflammation. In the present study, we conducted a randomized clinical trial that recruited adults with fasting serum low HDL-C to assess the effects of three-month supplementation with capsaicin capsules on serum HDL-C and other lipids, lipoproteins, and apolipoproteins levels. Additionally, the effects of capsaicin on HDL-C metabolism and several inflammatory cytokines were also estimated.

## 2. Methods

### 2.1. Populations

Forty-two volunteers with low serum HDL-C levels were recruited between June 2014 and December 2014 from the population that entered the Southwest Hospital, Chongqing, China, for regular health examination. Before being enrolled in the trial, all participants finished a physical examination and medical history investigation in the hospital. The diagnostic criterion of low serum HDL-C level was set as a fasting serum HDL-C lower than 1.03 mmol/L for men or 1.30 mmol/L for women, according to the sex-specific values recommended by the National Cholesterol Education Program (NCEP) Expert Panel on Detection, Evaluation, and Treatment of High Blood Cholesterol in Adults (Adult Treatment Panel III) guidelines. The inclusion criteria were adults aged between 30 and 50 years, and having a steady body mass index (BMI) between 20 and 30 over the last three months. The exclusion criteria included the use of any medicine or dietary supplementation that could influence glucose and lipid metabolism in the last six months, severe chronic disease, liver or kidney dysfunctions or malignant tumors, any acute or chronic infectious disease, injury, or any surgical procedure.

### 2.2. Study Design

This study was a randomized, double-blind, controlled trial. Forty-two eligible people were randomly assigned to the control or to the capsaicin group and were asked to consume two control or capsaicin capsules twice daily for three months. The randomized sequence was produced by a randomization protocol using the IBM SPSS Statistics 18.0 system (IBM Corporation, New York, NY, USA) conducted by another person who did not participate in this trial, and the information of randomization was sealed until the end of the study. All participants and people who conducted the trial and assessed outcomes were blinded to the intervention information. All participants were asked to avoid consuming any chili or pepper and to maintain their other habitual dietary style and physical activities during the trial. Each participant visited the hospital at zero and three months, and fasting blood samples were collected for measuring the concentration of parameters such as lipids, glucose, and liver enzymes. Additionally, the height, weight, and blood pressure of each participant were measured at baseline.

The protocol was approved by the Medical Ethics Committee of Third Military Medical University on 13 March 2013. All participants provided written informed consent for inclusion before they participated in the study. All procedures were in accordance with institutional guidelines and were carried out in compliance with the Helsinki Declaration. This trial was conducted according to “good clinical practice”. The registered number of this trial was ChiCTR-TRC-14004605 in the Chinese clinical trial registry (http://www.chictr.org.cn).

### 2.3. Interventions

Pod pepper and Henan small pepper were produced by Chongqing Academy of Agricultural Sciences and Vegetables in China. The contents of capsaicin were 4 mg/g in the skin of pod pepper and 0.05 mg/g in the skin of the Henan small pepper, respectively, which were determined by using the high performance liquid chromatography (HPLC) method. The peppers then were manufactured into capsules in the department of pharmacy of Southwest Hospital in Chongqing, China. After deleting seeds and superfine treatment, the powder of the skin of pepper was manufactured into capsules with dextrin added. The contents of capsaicin in the two kinds of capsules were 1 mg (capsaicin capsule) and 0.0125 mg (control capsule), respectively. All capsules were visually identical and packaged in sealed white bottles. 

The participants in the capsaicin group consumed the capsaicin capsules, and the participants in the control group consumed the control capsules. To avoid stimulation of the gastrointestinal tract, all subjects were instructed to consume two capsules twice daily during lunch and dinner. Thus, the daily total intervention doses of capsaicin were 4 mg and 0.05 mg in the capsaicin and control groups. The dose of capsaicin used in the current trial was determined based on previous human studies and our recent study [10,14,15,16]. Our previous study found that a four-week intervention of 5 mg of capsaicin daily effectively ameliorated the postprandial hyperglycemia and hyperinsulinemia in women with gestational diabetes mellitus [10]. In a human study, the daily intake of 33 mg of capsaicin for four weeks was shown to be safe and effective for improving the attenuated postprandial hyperinsulinemia, fasting lipids, and inflammatory response in healthy humans [15].

### 2.4. Experimental Assays

#### 2.4.1. Anthropometric Measurements

Body weight was measured using electronic scales, with subjects wearing light clothing. Height was measured using a stadiometer. Blood pressure was measured with an Omron digital sphygmomanometer (Model T9P; OMRON Corporation, Kyoto, Japan).

#### 2.4.2. Serum Lipids, Lipoproteins, Apolipoproteins, and Glucose Assays

The primary outcomes were serum fasting HDL-C levels. Assessments of levels of serum fasting TC, triglycerides (TG), HDL-C and LDL-C, apolipoprotein AI (ApoAI) and B (ApoB), lipoprotein a (Lp_(a)_), and glucose were performed using routine methods in an automatic biochemical analyzer (Beckman, AU5800, Brea, CA, USA) [17]. Serum apolipoprotein E (apoE) levels were assessed using the immunoassay method (Ningbo Ruiyuan Biotechnology Co., Ltd., Ningbo, China) in the analyzer as described above. All of the above variables were measured at baseline and at three months. The within- and between-assay coefficient of variations (CVs) of the above methods were <5%.

#### 2.4.3. Serum Liver Enzymes, Kidney Parameters, and Blood Routine Assays

Serum levels of albumin, globulin, total protein, alkaline phosphatase (ALP), alanine aminotransferase (ALT), aspartate transaminase (AST), γ-glutamyltranspeptidase (GGT), urea nitrogen, uric acid, and creatinine were assessed using routine methods in an automatic biochemical analyzer (Beckman, AU5800, Brea, CA, USA) [17]. Blood routine examination was determined with a Blood Cell Analyzer (MEK-6318K; Nihon Kohden Corporation, Tokyo, Japan).

#### 2.4.4. CETP, LCAT, and PLTP Assays

Plasma activities of CETP, LCAT, and PLTP were assessed using fluorescence assay kits (Roar Biomedical, Inc., New York, NY, USA) with the fluorescence spectrophotometer SpectraMax M2 (Molecular Devices, LLC, Sunnyvale, CA, USA). The procedures of plasma CETP, PLTP, and LCAT activity measurements were according to the manufacturers’ instructors, and the procedures and mechanisms of the assays were described by others [18,19]. The assay of LCAT activity was based on the ratio of the levels of the hydrolyzed (390-nm peak) to the non-hydrolyzed (470-nm peak) phosphatidylcholine (as the substrate reagent). After the hydrolysis of phosphatidylcholine by plasma LCAT, there was an increase in the 390-nm emission with a decrease of the 470-nm emission peak. Results are expressed as a ratio of 390/470 emitters, and the higher ratio presented the greater activity of LCAT. Serum concentrations of CETP and LCAT were detected using enzyme-linked immunosorbent assay (ELISA) kits (Shanghai Enzyme-linked Biotechnology Co., Shanghai, China) according to the manufacturers’ protocols with the same spectrometer. Thewithin- and between-assay CVs were <10.0% for activity assays and <7.2% for ELISA assays.

#### 2.4.5. Inflammatory Cytokine Assays

Plasma C-reactive protein (CRP) levels were assessed using the latex-enhanced immunoassay method (Nanjing Jiancheng Bioengineering Institute, Nanjing, China). Serum levels of serum amyloid A (SAA) and tumor necrosis factor-α (TNF-α) were detected using ELISA kits (Shanghai Enzyme-linked Biotechnology Co., Ltd., Shanghai, China). All measurements were performed according to the manufacturers’ protocol with the above spectrometer, and the within- and between-assay CVs were <6.7%.

### 2.5. Statistical Analyses

The primary endpoint was the change from baseline (three months—baseline) in the fasting serum HDL-C. The present trial was designed to provide a greater than 80% statistical power to determine at 0.1 mmol/L reduction in fasting serum HDL-C after three months of the capsaicin capsule treatment compared with the control capsule intervention. The Standard Deviation (SD) of the effects of both capsules’ supplementation was set at 0.1 mmol/L. The significance level was set at 0.05, and two-tailed tests were used. It was estimated that a sample size of 42 was sufficient to test the primary triglyceride hypothesis while allowing for a 20% dropout rate.

Per protocol statistical method was used. All statistical analyses were performed using SPSS Version 18.0 (SPSS Inc., Chicago, IL, USA). Normally distributed data were expressed as the means ±SDs. Data that were not normally distributed were expressed as the median with the interquartile range and analyzed after logarithmic transformation. Student’s independent *t*-test was used for comparing the levels of anthropometric parameters and all serum or plasma parameters at baseline between the two groups. The difference in gender between the two groups was assessed by chi-square tests. The change in each parameter was calculated as the difference between the end value and the baseline value. The difference in the change of serum parameters between the two groups was tested by Student’s independent *t*-test. The effects of the capsaicin supplementations on serum parameters compared with the control supplementations were analyzed using one-factor analysis of covariance (ANCOVA) with the change in each parameter as the dependent variable, the treatment variable as an independent variable, and adjusted with the corresponding values at baseline (Model 1) or adjusted with the corresponding values at baseline, age, gender, and BMI (Model 2, as the primary analysis). The reported *p*-value (for each outcome variable) in ANCOVA was the *p*-value associated with the treatment variable. Two-sided *p*-values < 0.05 were considered significant.

## 3. Results

### 3.1. Characteristics of the Participants

**Figure 1:**
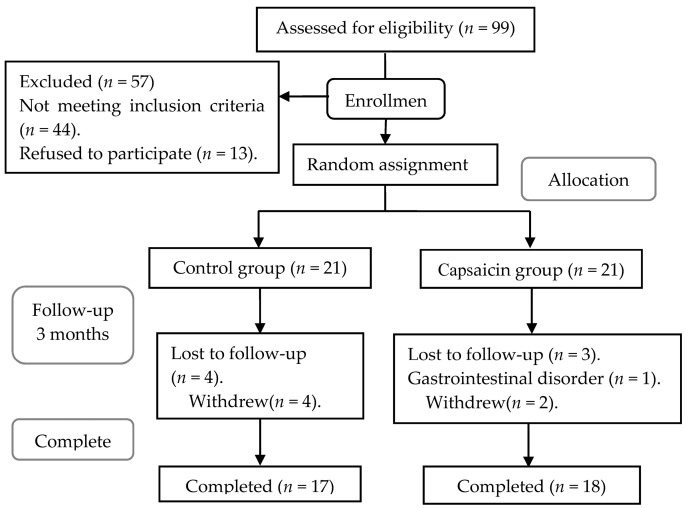
Flow of subjects throughout the study.

The baseline characteristics of the participants in the two groups were similar (Table 1). After the three-month intervention, 35 subjects (17 and 18 subjects in the control and the capsaicin groups, respectively) completed this trial (Figure 1).

### 3.2. Compliance

Seven subjects withdrew from the study, for reasons including unacceptable stimulation of the gastrointestinal tract (one person in the capsaicin group), refused to go back (three persons), and disconnect (three persons). No adverse reactions were reported by the participants during the trial. The compliance of all subjects was good by self-report and capsule counts. The rate of capsules consumed was 97.9% in the control group and 97.2% in the capsaicin group.

### 3.3. Effects of Capsaicin on the Serum Lipids, Lipoprotein, Apolipoproteins, and Glucose of the Participants

Compared to the control intervention, fasting serum HDL-C levels of the participants increased, while TG levels and the ratio of total cholesterol to HDL-C decreased significantly after capsaicin intervention for three months (Table 2). There was no significant difference between the effects of capsaicin and control supplementations on fasting serum total cholesterol, LDL-C, non-HDL-C, Lp_(a)_, ApoAI, ApoB, ApoE, and glucose. Adjustments for age, gender, and BMI at baseline did not alter these results.

### 3.4. Effects of Capsaicin on the Blood CETP, LCAT, and PLTP of the Participants

After three months of intervention, plasma PLTP activities decreased more in the capsaicin group than in the control group (*p* = 0.043, Table 3). However, serum concentrations and activities of CETP and LCAT did not significantly change between the two groups. These results did not alter after adjustments for age, gender, and BMI at baseline.

### 3.5. Effects of Capsaicin on the Serum Liver, Kidney Parameters, and Blood Routine of the Participants

Compared to the control intervention, serum creatinine increased in the capsaicin group compared to in the control group, while the creatinine levels at the end were still normal. Serum liver parameters, urea nitrogen, and uric acid concentrations and blood routine examination parameters in both groups did not significantly change after the three-month trial (Table 4). Additionally, the blood routine examination parameters did not differ in either group during the trial (data not shown).

### 3.6. Effects of Capsaicin on the Inflammatory Cytokines of the Participants

Compared to the control group, capsaicin intervention for three months significantly reduced plasma CRP levels in patients with lower HDL-C levels (Table 5). Serum SAA and TNF-α levels did not significantly change during the trial in the two groups.

## 4. Discussion

To our knowledge, few clinical trials focused on the treatment of HDL-C were conducted in adults with low HDL-C, and the major drugs used were niacin, statins, and cholesteryl ester transfer protein inhibitors. Our study was the first clinical trial using a novel phytochemical capsaicin for the treatment of low HDL-C, conducted in adults with low HDL-C. In the current study, daily administration of 4 mg of capsaicin for three months effectively increased the levels of fasting serum HDL-C combined with the inhibition of PLTP activity and anti-inflammation in the adults with low HDL-C. Additionally, capsaicin also lowered the levels of TG and the ratio of TC to HDL-C, while TC, LDL-C, apolipoproteins, Lp_(a)_, glucose, as well as concentrations and activities of LCAT and CETP did not differ during the trial. 

Current pharmaceutical interventions such as niacin and torcetrapib significantly increased the levels of HDL-C, but did not affect the development of cardiovascular diseases (CVD) [20,21]. Thus, novel approaches to increase the HDL-C levels and prevent CVD and other metabolic diseases are needed. From the above perspective, our finding was exciting. So far, few clinical trials have reported the influence of capsaicin on HDL-C and other lipids. Among these studies, the populations were healthy volunteers or women with gestational diabetes mellitus [10,15,22,23], and the levels of HDL-C of these people were generally normal. The above studies found no obvious influence of capsaicin supplementations on the HDL-C concentration [10,15,22,23]. In the current study, capsaicin effectively increased serum fasting HDL-C levels by about 0.08 mmol/L (3 mg/dL) in individuals with low HDL-C. An increase of about 3 mg/dL of HDL-C is clinically relevant. To put this into perspective, it has been reported that every 1-mg/dL increment in HDL-C levels is related to reductions of 2–3% in CVD risk [24]. Therefore, the 3-mg/dL increase in HDL-C observed in the current study would result in a nearly 9% reduction in CVD risk. However, the beneficial effects of capsaicin on the prevention and treatment of CVD need to be confirmed in larger and longer clinical investigations or with higher doses if necessary in the future.

The observed changes in HDL are noteworthy because the capsaicin capsules contained powders of the skin of chili. Besides the populations in Chongqing, China, in the current study, many populations in many countries such as Mexico, Argentina, Malaysia, South Korea, and India and some Americans and British are in favor of food containing chili or capsaicin. Additionally, the intervention dose of capsaicin in the current trial was 4 mg, which is lower than the doses used in other studies [16,25]. Further investigation is required to assess whether a higher intervention dose of capsaicin leads to a greater HDL-C increase.

Additionally, the reduction effect of capsaicin on the TG concentration observed in the current study was similar to our previous study, which was conducted in women with gestational diabetes mellitus [10]. Consumption of capsaicin-containing chili powder for four weeks significantly decreased mean TG levels from 3.67 mmol/L down to 2.75 mmol/L in these women, who often had higher levels of fasting serum TG [10]. However, another clinical study conducted in healthy humans reported that a capsaicin-containing chili intervention for four weeks did not affect lipid metabolism [16]. Based on the different findings between the above study and our studies, we assumed that consumptions of capsaicin or chili may attenuate lipid disorders, while not affecting normal lipid metabolism.

In addition, the present study demonstrated that capsaicin supplementations significantly decreased the activity of PLTP, while the concentrations and activities of CETP and LCAT did not differ. To the best of our knowledge, previous studies reported that neither food or a food component nor drugs such as statins and bezafibrate could influence the activity of PLTP in humans [26,27]. The current study was the first to report that capsaicin (a food component) effectively reduced the activity of PLTP. Elevated plasma PLTP activity is considered as a novel marker of cardiovascular disease susceptibility [28], and is positively associated with left ventricular systolic dysfunction in patients with CHD [29] and the carotid intima-media thickness in type 2 diabetes mellitus [30]. Some studies found that plasma PLTP activity was inversely correlated with HDL-C levels in humans with coronary heart disease (CHD) or premenopausal women [31,32]. However, we did not find any relationship between the effects of capsaicin on the increment of HDL-C with the reduction of PLTP activity by Pearson correlation and partial correlation analyses adjusted for age, gender, and BMI (data not shown). PLTP activity was positively related to large HDL particles in patients with type 2 diabetes mellitus [33] and positively correlated with the concentration of HDL particles containing Apo AI, but not Apo AII in patients with low HDL and CVD [34]. In the current study, we did not classify HDL particles and had no results on the relations of plasma PLTP activity and HDL particles. The mechanism underlying the protective actions of capsaicin on lipids and the role of PLTP warrant further research. Importantly, this study was the first to find healthful effects of capsaicin on HDL-C levels and PLTP activity in humans.

Finally, the current study observed that capsaicin consumption significantly decreased CRP levels in adults with low HDL-C, and suggested that capsaicin performed an anti-inflammatory effect in humans. This result was consistent with previous studies [6]. In general, inflammation is related to HDL-C metabolism [13]. We assumed that the beneficial effects of capsaicin supplementation on HDL-C levels and PLTP activity may be associated with its anti-inflammatory effect.

The healthful effects of capsaicin in adults with low HDL-C levels may be associated with the change of gut microbiota. Our previous animal studies reported that gut microbiota play a critical role in the beneficial effects of dietary capsaicin consumption against chronic low-grade inflammation and associated dietary-induced obesity [35]. NAFLD is generally associated with inflammation and obesity. We presumed that gut microbiota had participated in the protective effects of capsaicin supplementations in the increment of HDL-C and the reductions of TG and CRP in adults with low HDL-C in the current study. 

There were still some limitations in the present study. First, the above beneficial effects of capsaicin capsules in adults with low HDL-C were observed in a small sample. Our results should be confirmed in more clinical trials with larger sample sizes. Second, this study used capsaicin capsules as the intervention, which may lead to stimulation of the gastrointestinal tract. Although the gastrointestinal tract may adapt to capsaicin via uncertain mechanisms, such as the change of gastrointestinal microflora [36], the applications of capsaicin capsules may still be limited. Capsiate, an analogue of capsaicin that has not been studied in humans, may demonstrate many actions similar to capsaicin [37,38]. However, a previous study found that capsaicin, not capsiate, had the abilities of reducing plasma cholesterol and inhibiting the formation of atherosclerotic plaque in hamsters [7]. Thus, whether capsiate has the same effect as capsaicin on low HDL-C adults should be studied in the future. In addition, the use of the sustained release method may help to reduce the stimulation of the gastrointestinal tract induced by capsaicin. Third, adults with low HDL-C only consumed capsaicin capsules for three months in the current study. Although the fasting serum HDL-C levels of participants significantly increased after the three-month intervention of capsaicin, the fasting serum HDL-C levels were still lower than normal levels at the end of the trial. A longer intervention duration or a higher dose of capsaicin may exert a better effect on adults with low HDL-C. Finally, there were fewer women with low HDL-C recruited in the trial, so the effects of capsaicin on women with low HDL-C should be confirmed in other clinical trials in the future.

## 5. Conclusions

In conclusions, three-month supplementations with capsaicin capsules, containing powders of chili skin, significantly increased fasting serum HDL-C levels and decreased serum TG levels, combined with the inhibition of PLTP activity and the anti-inflammatory effect in adults with low HDL-C levels. 

## Figures and Tables

**Table 1:** The characteristics of the study participants at baseline.^1^

Variables	Control (*n* = 17)	Capsaicin (*n* = 18)	*p*
Age (years)	45.2 ± 4.8	41.9 ± 5.7	0.07
Male/Female (*n*)	14/3	16/2	0.66
Height (cm)	164.5 ± 7.4	166.6 ± 6.6	0.40
Weight (kg)	70.4 ± 7.9	73.9 ± 11.7	0.30
BMI (kg/m^2^)	26.03 ± 2.95	26.46 ± 2.76	0.65
SBP (mmHg)	128.8 ± 17.0	123.1 ± 13.7	0.29
DBP (mmHg)	84.9 ± 11.6	83.7 ± 10.9	0.76
HDL-C (mmol/L)	0.88 ± 0.13	0.92 ± 0.13	0.38

^1^ Values are presented as the means ±SDs or *n*. Abbreviations: DBP, diastolic blood pressure; SBP, systolic blood pressure.

**Table 2:** Effects of capsaicin on the serum lipids, lipoprotein, apolipoproteins, and glucose in the participants ^1^.

Variables	Control (*n* = 17)			Capsaicin (*n* = 18)					
	0 Month	3 Months	Change	0 Month	3 Months	Change	*P_t_*	*P_M1_*^2^	*P_M2_*^3^
TC (mmol/L)	4.28 ± 0.87	4.73 ± 1.27	0.45 ± 1.04	4.56 ± 0.67	4.61 ± 1.16	0.05 ± 0.93	0.30	0.28	0.12
LDL-C (mmol/L)	2.42 ± 0.55	2.33 ± 0.42	−0.09 ± 0.46	2.46 ± 0.44	2.50 ± 0.54	0.04 ± 0.37	0.83	0.27	0.48
HDL-C (mmol/L)	0.88 ± 0.13	0.86 ± 0.13	−0.02 ± 0.14	0.92 ± 0.13	1.00 ± 0.13	0.08 ± 0.14	0.38	0.005	0.030
non-HDL-C (mmol/L)	3.40 ± 0.80	3.88 ± 1.3	0.47 ± 1.03	3.64 ± 0.68	3.61 ± 1.13	−0.03 ± 0.91	0.35	0.15	0.08
TC/HDL-C	4.88 ± 0.79	5.72 ± 2.36	0.84 ± 1.92	5.06 ± 1.13	4.64 ± 1.12	−0.42 ± 1.13	0.59	0.027	0.043
TG (mmol/L)	2.91 (1.69, 3.88)	2.38 (1.65, 4.45)	0.29 (−0.39, 1.03)	2.62 (1.91, 5.57)	2.05 (1.43, 3.57)	−0.65 (−1.71, 0.04)	0.66	0.019	0.027
Lp_(a)_ (mg/L)	61.0 (26.5, 200.5)	60.0 (32.5, 134.5)	0.0 (−33.0, 14.5)	33.0 (21.25, 74.75)	26.0 (14.25, 56.25)	−8.5 (−19.25, 5.25)	0.23	0.34	0.15
ApoAI (g/L)	1.09 ± 0.14	1.11 ± 0.11	0.02 ± 0.11	1.11 ± 0.17	1.12 ± 0.10	0.01 ± 0.19	0.70	0.86	0.82
ApoB (g/L)	0.96 ± 0.19	0.90 ± 0.19	−0.06 ± 0.19	0.90 ± 0.18	0.90 ± 0.17	−0.01 ± 0.17	0.34	0.63	0.76
ApoE (mg/dL)	5.03 ± 1.55	6.55 ± 4.25	1.52 ± 3.21	5.74 ± 2.85	5.43 ± 3.14	−0.32 ± 2.97	0.37	0.11	0.16
Glucose (mmol/L)	5.27 ± 0.41	5.30 ± 0.38	0.03 ± 0.46	5.49 ± 2.24	5.47 ± 1.71	−0.02 ± 0.68	0.70	0.97	0.64

^1^ Values are presented as the means ± SDs or medians (interquartile range). Abbreviations: ApoAI, apolipoproteinAI; ApoB, apolipoprotein B; ApoE, apolipoprotein E; HDL-C, high-density lipoprotein cholesterol; LDL-C, low-density lipoprotein cholesterol;Lp_(a)_, lipoproteina; TC, total cholesterol; TG, triglycerides. ^2^ Model 1: one-factor analysis of covariance (ANCOVA), with the change at three months from baseline considered as the dependent variable and the baseline value considered as the covariate.^3^ Model 2: Model 1 adjusted for the corresponding values at baseline, age, gender, and BMI.

**Table 3:** Effects of capsaicin on the blood CETP, LCAT, and PLTP in the participants^1^.

Variables	Control (*n* = 17)			Capsaicin (*n* = 18)					
	0 Month	3 Months	Change	0 Month	3 Months	Change	*P_t_*	*P_M1_*^2^	*P_M2_*^3^
CETP concentration (mg/L)	2.02 ± 0.89	2.37 ± 1.15	0.35 ± 0.88	2.07 ± 1.06	2.30 ± 1.07	0.22 ± 0.94	0.87	0.71	0.92
CETP activity (mmol/h)	73.2 ± 8.34	73.14 ± 7.9	−0.06 ± 8.97	72.91 ± 9.72	71.61 ± 10.94	−1.3 ± 11.75	0.93	0.67	0.99
LCAT concentration (mg/L)	6.27 ± 3.73	7.38 ± 4.12	1.10 ± 2.55	7.25 ± 4.89	7.67 ± 4.46	0.42 ± 3.28	0.51	0.65	0.67
LCAT activity (390/470 nm)	0.94 ± 0.07	0.97 ± 0.11	0.03 ± 0.12	0.95 ± 0.18	0.95 ± 0.09	0.01 ± 0.15	0.80	0.66	0.73
PLTP activity (mmol/h)	1.52 ± 0.38	1.64 ± 0.42	0.12 ± 0.39	1.67 ± 0.39	1.44 ± 0.32	−0.22 ± 0.4	0.27	0.030	0.043

^1^ Values are presented as the means ± SDs. Abbreviations: CETP, cholesteryl ester transfer protein; LCAT, lecithin cholesterol acyltransferase; PLTP, phospholipid transfer protein. ^2^ Model 1: one-factor ANCOVA, with the change at three months from baseline considered as the dependent variable and the baseline value considered as the covariate. ^3^ Model 2: Model 1 adjusted for the corresponding values at baseline, age, gender, and BMI.

**Table 4:** Effects of capsaicin on the serum liver and kidney parameters in the participants^1^.

Variables	Control (*n* = 17)			Capsaicin (*n* = 18)					
	0 Month	3 Months	Change	0 Month	3 Months	Change	*P_t_*	*P_M1_*^2^	*P_M2_*^3^
Albumin(g/L)	45.7 ± 1.6	45.9 ± 2.4	0.2 ± 2.5	46.7 ± 3.0	46.0 ± 2.7	−0.7 ± 2.7	0.23	0.64	0.43
Globulin (g/L)	29.1 ± 3.1	29.9 ± 2.9	0.8 ± 3.4	29.4 ± 2.7	29.8 ± 2.6	0.3 ± 3.2	0.73	0.78	0.83
Total protein (g/L)	74.8 ± 3.4	76.0 ± 3.4	1.2 ± 4.0	76.1 ± 4.1	75.8 ± 3.4	−0.3 ± 4.0	0.31	0.57	0.63
A/G ratio	1.59 ± 0.17	1.55 ± 0.17	−0.04 ± 0.21	1.60 ± 0.17	1.56 ± 0.18	−0.04 ± 0.2	0.83	0.97	0.66
GGT (IU/L)	22.0 (20.5, 52.5)	21.0 (17.0, 45.3)	3.0 (1.0, 9.0)	29.0 (24.5, 116.5)	25.5 (18.8, 45.8)	4.0 (−1.5, 7.25)	0.16	0.54	0.30
ALT (IU/L)	26.9 ± 19.4	27.1 ± 9.9	0.1 ± 15.7	23.3 ± 7.8	27.2 ± 10.3	3.9 ± 7.2	0.46	0.58	0.59
AST (IU/L)	24.1 ± 7.6	24.3 ± 3.6	0.2 ± 7.8	22.3 ± 4.2	24.9 ± 5.9	2.6 ± 5.2	0.39	0.54	0.87
ALP (IU/L)	94.4 ± 31.8	95.7 ± 25.0	1.4 ± 17.3	81.8 ± 18.8	79.6 ± 18.4	−2.3 ± 12.2	0.16	0.10	0.11
Creatinine (μmol/L)	74.46 ± 11.93	74.48 ± 13.20	0.02 ± 6.08	75.3 ± 13.15	81.46 ± 12.50	6.16 ± 10.22	0.85	0.029	0.015
Urea nitrogen (mmol/L)	5.04 ± 1.08	5.39 ± 1.35	0.35 ± 1.17	5.06 ± 1.65	5.13 ± 1.34	0.07 ± 1.37	0.97	0.49	0.20
Uric acid (μmol/L)	378.0 ± 76.4	406.6 ± 77.5	28.7 ± 50.1	390.7 ± 88.9	407.1 ± 91.9	16.4 ± 45.1	0.65	0.52	0.68

^1^ Values are presented as the means ± SDs or medians (interquartile range). Abbreviations: A/G ratio, albumin/globulin ratio; ALP, alkaline phosphatase; ALT, alanine aminotransferase; AST, aspartate transaminase; GGT, γ-glutamyl transpeptidase.^2^ Model 1: one-factor ANCOVA, with the change at three months from baseline considered as the dependent variable and the baseline value considered as the covariate. ^3^ Model 2: Model 1 adjusted for the corresponding values at baseline, age, gender, and BMI.

**Table 5:** Effects of capsaicin on the inflammatory cytokines in the participants^1^.

Variables	Control (*n* = 17)			Capsaicin (*n* = 18)					
	0 Month	3 Months	Change	0 Month	3 Months	Change	*P_t_*	*P_M1_*^2^	*P_M2_*^3^
CRP (μg/mL)	1.67 (1.10, 2.36)	1.89 (1.39, 2.76)	0.24 (−0.73, 1.04)	1.38 (1.13, 2.03)	1.09 (0.83, 1.74)	−0.39 (−1.08, 0.26)	0.69	0.034	0.042
TNF-α (pg/mL)	31.49 ± 14.75	35.04 ± 16.85	3.55 ± 13.41	33.42 ± 18.11	34.92 ± 15.59	1.49 ± 15.65	0.73	0.78	0.95
SAA (μg/mL)	4.08 ± 1.64	4.73 ± 2.28	0.66 ± 1.89	4.69 ± 3.04	5.34 ± 3.30	0.65 ± 2.75	0.46	0.83	0.88

^1^ Values are presented as the means ± SDs or medians (interquartile range). Abbreviations: CRP, C-reactive protein; SAA, serum amyloid A; TNF-α, tumor necrosis factor-α. ^2^ Model 1: one-factor ANCOVA, with the change at three months from baseline considered as the dependent variable and the baseline value considered as the covariate. ^3^ Model 2: Model 1 adjusted for the corresponding values at baseline, age, gender, and BMI.
